
JOURNAL INFORMATION
==============================
NLM Title Abbreviation: Virus Evol
Journal ID: Virus Evol
NLM Journal ID: 101664675
Journal ID: vevolu

Virus Evolution

EISSN: 2057-1577

ARTICLE INFORMATION
==============================
PMCID: PMC8195028
PMID: 34527283
DOI: 10.1093/ve/veab053
Article ID: veab053
Article version: 1
Subjects: Reflections, AcademicSubjects/MED00860, AcademicSubjects/SCI01130, AcademicSubjects/SCI02285

Coordinating SARS-CoV-2 Genomic Surveillance in the United States

https://orcid.org/0000-0003-4814-0179 Nelson Martha I Laboratory of Parasitic Diseases, Division of Intramural Research, National Institute of Allergy and Infectious Diseases, National Institutes of Health, Bethesda, MD USA

https://orcid.org/0000-0003-1807-2785 Thielen Peter Biological Sciences Group, Research and Exploratory Development Department, Johns Hopkins University Applied Physics Laboratory, Laurel, MD USA

Corresponding author: Martha I. Nelson, 50 South Drive, Building 50 Room 1505, Bethesda, MD 20814; nelsonma@mail.nih.gov; 301-761-7831
Electronic publication date: 2021 Jun 5
Electronic Location ID: veab053
Received 2021 Apr 2; Revised 2021 May 21; Accepted 2021 Jun 4; 2021 Jun 2
Copyright: Published by Oxford University Press 2021. This work is written by (a) US Government employee(s) and is in the public domain in the US.
Copyright year: 2021
License: This article is made available via the PMC Open Access Subset for unrestricted re-use and analyses in any form or by any means with acknowledgement of the original source. These permissions are granted for the duration of the COVID-19 pandemic or until permissions are revoked in writing. Upon expiration of these permissions, PMC is granted a perpetual license to make this article available via PMC and Europe PMC, consistent with existing copyright protections.
License: This work is written by (a) US Government employee(s) and is in the public domain in the US.
License URL: https://creativecommons.org/licenses/by/4.0/

Funding: Division of Intramural Research, National Institute of Allergy and Infectious Diseases 10.13039/100006492
edited-state: accepted-manuscript
article-lifecycle: PAP

==============================
### Coordinating SARS-CoV-2 Genomic Surveillance in the United States

Martha I. Nelson[1§] and Peter Thielen[2]

[1] Laboratory of Parasitic Diseases, Division of Intramural Research, National Institute of Allergy and Infectious Diseases, National Institutes of Health, Bethesda, MD USA

[2] Biological Sciences Group, Research and Exploratory Development Department, Johns Hopkins University Applied Physics Laboratory, Laurel, MD USA

Word Count: 1,755

[§]Corresponding author: Martha I. Nelson, 50 South Drive, Building 50 Room 1505, Bethesda, MD 20814; nelsonma@mail.nih.gov; 301-761-7831

ACCEPTED MANUSCRIPT

The United States has rapidly responded to the emergence of new SARS-CoV-2 variants of concern (VOC) by scaling up genomic surveillance. Tens of thousands of viral genomes are now sequenced in American labs each week to track the spread of variants originating in the US (Annavajhala et al. 2021; Deng et al. 2021) or imported from other countries (Washington et al. 2021) to keep diagnostics, therapeutics, and vaccines up to date (Walensky et al., 2021). An influx of Federal funding provides an unprecedented opportunity to build a new US genomic surveillance system from the ground up, informed by in-country expertise (National Academies of Sciences 2020; Black et al. 2020) as well as existing models of successful genomic surveillance systems established in other countries (COVID-19 Genomics UK 2020; Seemann et al. 2020; Msomi et al. 2020). Fully leveraging genetic data requires a centrally coordinated national sampling strategy and consortiums for sharing valuable metadata, which are needed to study how new variants evade host immunity, cause severe disease, or transmit differently in human populations. However, US public and private labs have a history of autonomy and strong protections for patient privacy, presenting ongoing barriers to central coordination and data sharing.

© Published by Oxford University Press 2021. This work is written by (a) US Government employee(s) and is in the public domain in the US.

**Centrally Coordinated Sampling Strategy.** Routine, population-based sampling that provides an unbiased, representative survey of the genetic composition of viruses circulating over time and space is the gold standard for tracking how new variants relate to disease severity, population immunity, and epidemic trajectory. Instead, genomic surveillance is frequently performed opportunistically for practical reasons, introducing biases that limit the downstream utility of the data. America's vast network of public and private labs have independently generated large numbers of SARS-CoV-2 genomes that provide highly resolved pictures of the genetic diversity and transmission chains underlying local epidemics (Gonzalez-Reiche et al. 2020; Chu et al. 2020; Lemieux et al. 2021; Bedford et al. 2020). But these pursuits often target local or hospital-based populations with detailed patient metadata, and do not always align with broader population-based surveillance of national interest. Coordinating the efforts of America's diverse network of state, commercially run, and academic labs within a nationwide surveillance consortium that standardizes population-based sampling is no small feat, but the success of the genomics program hinges on it. Carrots work better than sticks and one reason the UK consortium has been successful is because participants access user-friendly, customizable tools for visualizing local and national data trends over time and space (Nicholls et al. 2020; Argimón et al. 2016). Both cloud-hosted and locally implemented bioinformatics tools enable quick conversion from unprocessed sequence data to deposition in global data platforms (Grubaugh et al. 2019; Connor et al. 2016; Singer et al. 2020; Rambaut et al. 2020). Local officials also see benefits when real-time genomic data explain the necessity of unpopular policy reversals, such as the school closures that followed the spike of highly transmissible B.1.1.7 variants in the UK in December 2020 (Volz et al. 2021).

**Building a Research Network for Genomic Epidemiology.** High throughput bioinformatic pipelines allow state and local public health labs to flag new VOCs emerging in communities

ACCEPTED MANUSCRIPT

(Hadfield et al., 2018). But fully leveraging genetic data to understand variants' epidemiological impact requires expertise in advanced phylodynamic methods that are still far from being automated (Lemey et al. 2020; du Plessis et al. 2021). Years ago, the Federal government had the foresight to establish two highly successful research networks, RAPIDD (Research and Policy for Infectious Disease Dynamics) and MIDAS (Models of Infectious Disease Agent Study), with experts in infectious disease epidemiology and modeling across academia and government (Nelson et al. 2019). The return on investment was high during outbreaks of Ebola, Zika, and pandemic influenza, when epidemiological expertise was on hand to guide vaccination strategies and other countermeasures (Merler et al. 2016). Moreover, the networks had a major downstream impact on developing a new workforce of talented young epidemiologists capable of dealing with highly complex epidemiological data for public health. The highly collaborative alumni network has continued to drive the science behind COVID-19 vaccine strategies, mask wearing, school closures, and social distancing (Borchering et al. 2021). Establishing a similar network for genomic epidemiology would ensure that material investments in generating genomic raw data translate into evidence-based guidance.

**Patient Privacy**. A pervasive barrier for US researchers tracking SARS-CoV-2 variants has been the difficulty linking a genomic sequence to contextual information about the patient (Black et al. 2020). Most SARS-CoV-2 sequences submitted to public repositories include accurate information about the US state and date (month, day, year) of collection, as well as patient age and sex. However, clinicians, public health labs, and academic researchers routinely collect more detailed information, including the patient's county or zip code of residence, travel history, disease severity, as well as contact tracing data. Such information is vital to answer fundamental questions about how new variants transmit and cause disease across different ages (Davies et al. 2021)? However, sharing patient data publicly or with a collaborating research group in the US is often blocked, even when de-identified, due to strong protections for

ACCEPTED MANUSCRIPT

patient personal health information and informed consent requirements for samples used in research (Beach et al. 2020). Removing a patient's name does not necessarily achieve de-identification when there are few COVID-19 cases in a community, patient names are reported in the news or known within organizations, and detailed spatial-temporal information is included with a sequence.

Genomic epidemiology has only come of age recently and there has been little time to establish a regulatory framework. As a result there is no national consensus for what granularity of patient data can be released with a genetic sequence, leaving state and local health departments and Institutional Review Boards (IRB) to make decisions *ad hoc*. There are incentives to err on the side of caution when statistical uncertainty about identification combines with high penalties for violating the Health Insurance Portability and Accountability Act of 1996 (HIPAA) (United States 1996). Even within the National Institutes of Health information on virus genetics is intentionally separated from epidemiological information on patients. Protecting individuals is a primary concern, but barriers to integrating genomic and epidemiological data across labs limits statistical power and impedes US-wide analyses. One proposed solution is to establish a new US database restricted to a consortium of researchers and public health workers, similar to the UK (Nicholls et al. 2020), so that sensitive data can be shared but not released publicly (Maxmen 2021a). Sharing data in a central database also requires clarified HHS guidance on what level of patient data granularity can be shared in different contexts without violating federal law.

ACCEPTED MANUSCRIPT

**Standardized Metadata.** From a practical standpoint, overstretched public health workers do not necessarily have the time to submit dozens of metadata fields that are potentially of interest to researchers. Many fields require time-consuming manual extraction from patient charts. National priorities need to be predefined and streamlined to scale up data integration nationally (Gardner et al. 2020). For example, a study based on tens of thousands of sequences for which basic clinical data is available (e.g., asymptomatic, mild, hospitalized, death) along with simplified patient characteristics (age, sex, presence or absence of comorbidities) (Volz et al. 2020), is likely to have more power than a study with more detailed metadata (e.g., temperature, cough, ventilator use, diabetes) but only hundreds of sequences (Thielen et al. 2021).

**Global Data Sharing**. The speed with which SARS-CoV-2 viruses spread across the world, particularly as international travel normalizes, means that all countries, even those as large as the US, depend on quality genomic data from other countries to quickly identify and characterize new variants emerging globally and trace how they infiltrate the US. Reaching the milestone of one million SARS-CoV-2 genomes submitted to GISAID reflects recent advances in genomic sequencing and data sharing on a global scale. However, enormous volumes of data introduce bioinformatic challenges that test scalability. The need to balance tradeoffs between the free flow of information and protecting data contributors has become dire as the sheer volume of genomic data becomes unwieldy for individual labs and standard bioinformatics software (Van Noorden 2021). Providing more open access to the GISAID API would facilitate the mass flow of data into new high throughput bioinformatic tools and pipelines. Realistically, there needs to be a conceptual distinction between unethically using another group's data as the focus of a study versus including genomes from other countries as background data. The value of inclusion in a 30-page supplementary acknowledgment table listing thousands of submitters is less clear in the million-genomes era. However, developing countries that shared valuable SARS-CoV-2 sequence data with the global community have received few COVID-19 vaccine doses to date, fueling concerns about exploitation and disincentivizing any relaxation of existing protections on virological data for the foreseeable future (Maxmen 2021b).

**Raw Sequence Data**. Consensus genomes are the primary unit of viral genome surveillance. High throughput sequencing platforms generate hundreds- or thousands-fold excess data to

ACCEPTED MANUSCRIPT

extract this consensus using a ‘majority wins’ strategy. The raw sequence data underlying consensus genomes can provide valuable insights into ambiguous base calls as well as minority variants. However, raw sequence reads submitted to the NCBI’s Sequence Read Archive (SRA) represent only a tiny fraction of genomes available on GenBank or GISAID. The low number of submissions is due to the logistical complexity of transferring large amounts of data, minimal observed benefit by submitters focused on generation of consensus genomes, and concerns about submitting sequence reads that may contain host DNA. Messaging needs to be improved that SRA has reduced barriers to upload and includes a process to remove host DNA. Additionally, the pathogen genomics community would benefit greatly from further development and adoption of standardized informatics workflows that easily transition from large core facilities to small independent laboratories, which often have minimal informatics support. Reducing the time between sequencing and data dissemination has been a primary bottleneck for many US groups that are establishing sequencing at scale for the first time, and often analytical capabilities are not in place to enable groups to both generate consensus sequences and upload the raw data that was used to generate them.

**Conclusions**. As countries ramp up genomics capacity to track a rapidly evolving virus (Rockett et al. 2020; Oude Munnink et al. 2020; Hammer et al. 2021; Tegally et al. 2021; Geoghegan et al. 2020), the infrastructures built will hopefully outlast the current pandemic and improve outbreak response for decades to come. Domestically, the US faces unusual structural challenges that go deeper than funding, but all stakeholders are incentivized by new systems that save time in managing large data streams, approving IRBs, and providing useful information to guide decision making. If you build it, they will come.

**Acknowledgments.** This work was supported in part by the Intramural Research Program of the NIH.

ACCEPTED MANUSCRIPT

**Disclaimer**. The content does not necessarily reflect the views or policies of the U.S. Government or imply endorsement of any products.

**References**

Annavajhala, Medini K., Hiroshi Mohri, Jason E. Zucker, Zizhang Sheng, Pengfei Wang, Angela Gomez-Simmonds, David D. Ho, and Anne-Catrin Uhlemann. 2021. "A Novel SARS-CoV-2 Variant of Concern, B.1.526, Identified in New York." *medRxiv*, February. https://doi.org/10.1101/2021.02.23.21252259.

Argimón, Silvia, Khalil Abudahab, Richard J. E. Goater, Artemij Fedosejev, Jyothish Bhai, Corinna Glasner, Edward J. Feil, et al. 2016. "Microreact: Visualizing and Sharing Data for Genomic Epidemiology and Phylogeography." *Microbial Genomics* 2 (11): e000093.

Beach, Mary Catherine, Howard M. Lederman, Megan Singleton, Roy G. Brower, Joseph Carrese, Daniel E. Ford, Bhakti Hansoti, et al. 2020. "Desperate Times: Protecting the Public From Research Without Consent or Oversight During Public Health Emergencies." *Annals of Internal Medicine*, July. https://doi.org/10.7326/M20-4631.

Bedford, Trevor, Alexander L. Greninger, Pavitra Roychoudhury, Lea M. Starita, Michael Famulare, Meei-Li Huang, Arun Nalla, et al. 2020. "Cryptic Transmission of SARS-CoV-2 in Washington State." *Science*, September. https://doi.org/10.1126/science.abc0523.

Black, Allison, Duncan R. MacCannell, Thomas R. Sibley, and Trevor Bedford. 2020. "Ten Recommendations for Supporting Open Pathogen Genomic Analysis in Public Health." *Nature Medicine* 26 (6): 832–41.

Borchering, Rebecca K., Cécile Viboud, Emily Howerton, Claire P. Smith, Shaun Truelove, Michael C. Runge, Nicholas G. Reich, et al. 2021. "Modeling of Future COVID-19 Cases, Hospitalizations, and Deaths, by Vaccination Rates and Nonpharmaceutical Intervention Scenarios - United States, April-September 2021." *MMWR. Morbidity and Mortality Weekly Report* 70 (19): 719–24.

Bugembe, Daniel Lule, My V.T.Phan, Isaac Ssewanyana, Patrick Semanda, Hellen Nansumba, Beatrice Dhaala, Susan Nabadda, et al. 2021. "A SARS-CoV-2 Lineage A Variant (A.23.1) with Altered Spike Has Emerged and Is Dominating the Current Uganda Epidemic." *bioRxiv*. medRxiv. https://doi.org/10.1101/2021.02.08.21251393.

Chu, Helen Y., Janet A. Englund, Lea M. Starita, Michael Famulare, Elisabeth Brandstetter, Deborah A. Nickerson, Mark J. Rieder, et al. 2020. "Early Detection of Covid-19 through a Citywide Pandemic Surveillance Platform." *The New England Journal of Medicine* 383 (2): 185–87.

Connor, Thomas R., Nicholas J. Loman, Simon Thompson, Andy Smith, Joel Southgate, Radoslaw Poplawski, Matthew J. Bull, et al. 2016. "CLIMB (the Cloud Infrastructure for Microbial Bioinformatics): An Online Resource for the Medical Microbiology Community." *Microbial Genomics* 2 (9): e000086.

COVID-19 Genomics UK (COG-UK). 2020. "An Integrated National Scale SARS-CoV-2 Genomic Surveillance Network." *The Lancet. Microbe* 1 (3): e99–100.

Davies, Nicholas G., Christopher I. Jarvis, CMMID COVID-19 Working Group, W. John Edmunds, Nicholas P. Jewell, Karla Diaz-Ordaz, and Ruth H. Keogh. 2021. "Increased Mortality in Community-Tested Cases of SARS-CoV-2 Lineage B.1.1.7." *Nature*, March. https://doi.org/10.1038/s41586-021-03426-1.

Deng, Xianding, Miguel A. Garcia-Knight, Mir M. Khalid, Venice Servellita, Candace Wang, Mary Kate Morris, Alicia Sotomayor-González, et al. 2021. "Transmission, Infectivity, and Antibody

ACCEPTED MANUSCRIPT

Neutralization of an Emerging SARS-CoV-2 Variant in California Carrying a L452R Spike Protein Mutation." *medRxiv*, March. https://doi.org/10.1101/2021.03.07.21252647.
Faria, Nuno R., Thomas A. Mellan, Charles Whittaker, Ingra M. Claro, Darlan da S. Candido, Swapnil Mishra, Myuki A. E. Crispim, et al. 2021. "Genomics and Epidemiology of the P.1 SARS-CoV-2 Lineage in Manaus, Brazil." *Science*, April. https://doi.org/10.1126/science.abh2644.
Gardner, Lauren, Jeremy Ratcliff, Ensheng Dong, and Aaron Katz. 2020. "A Need for Open Public Data Standards and Sharing in Light of COVID-19." *The Lancet Infectious Diseases*, August. https://doi.org/10.1016/S1473-3099(20)30635-6.
Geoghegan, Jemma L., Xiaoyun Ren, Matthew Storey, James Hadfield, Lauren Jelley, Sarah Jefferies, Jill Sherwood, et al. 2020. "Genomic Epidemiology Reveals Transmission Patterns and Dynamics of SARS-CoV-2 in Aotearoa New Zealand." *Nature Communications* 11 (1): 6351.
Gonzalez-Reiche, Ana S., Matthew M. Hernandez, Mitchell J. Sullivan, Brianne Ciferri, Hala Alshammary, Ajay Obla, Shelcie Fabre, et al. 2020. "Introductions and Early Spread of SARS-CoV-2 in the New York City Area." *Science* 369 (6501): 297–301.
Grubaugh, Nathan D., Karthik Gangavarapu, Joshua Quick, Nathaniel L. Matteson, Jaqueline Goes De Jesus, Bradley J. Main, Amanda L. Tan, et al. 2019. "An Amplicon-Based Sequencing Framework for Accurately Measuring Intrahost Virus Diversity Using PrimalSeq and iVar." *Genome Biology* 20 (1): 8.
Hadfield, James, Colin Megill, Sidney M. Bell, John Huddleston, Barney Potter, Charlton Callender, Pavel Sagulenko, Trevor Bedford, and Richard A. Neher. 2018. "Nextstrain: Real-Time Tracking of Pathogen Evolution." *Bioinformatics* 34 (23): 4121–23.
Hammer, Anne Sofie, Michelle Lauge Quaade, Thomas Bruun Rasmussen, Jannik Fonager, Morten Rasmussen, Karin Mundbjerg, Louise Lohse, et al. 2021. "SARS-CoV-2 Transmission between Mink (Neovison Vison) and Humans, Denmark." *Emerging Infectious Diseases* 27 (2): 547–51.
Lemey, Philippe, Samuel L. Hong, Verity Hill, Guy Baele, Chiara Poletto, Vittoria Colizza, Áine O'Toole, et al. 2020. "Accommodating Individual Travel History and Unsampled Diversity in Bayesian Phylogeographic Inference of SARS-CoV-2." *Nature Communications* 11 (1): 5110.
Lemieux, Jacob E., Katherine J. Siddle, Bennett M. Shaw, Christine Loreth, Stephen F. Schaffner, Adrianne Gladden-Young, Gordon Adams, et al. 2021. "Phylogenetic Analysis of SARS-CoV-2 in Boston Highlights the Impact of Superspreading Events." *Science* 371 (6529). https://doi.org/10.1126/science.abe3261.
Maxmen, Amy. 2021a. "Massive Google-Funded COVID Database Will Track Variants and Immunity." *Nature*, February. https://doi.org/10.1038/d41586-021-00490-5.
———. 2021b. "Why Some Researchers Oppose Unrestricted Sharing of Coronavirus Genome Data." *Nature* 593 (7858): 176–77.
Merler, Stefano, Marco Ajelli, Laura Fumanelli, Stefano Parlamento, Ana Pastore Y Piontti, Natalie E. Dean, Giovanni Putoto, et al. 2016. "Containing Ebola at the Source with Ring Vaccination." *PLoS Neglected Tropical Diseases* 10 (11): e0005093.
Msomi, Nokukhanya, Koleka Mlisana, Tulio de Oliveira, and Network for Genomic Surveillance in South Africa writing group. 2020. "A Genomics Network Established to Respond Rapidly to Public Health Threats in South Africa." *The Lancet. Microbe* 1 (6): e229–30.
National Academies of Sciences, Engineering, and Medicine; Division on Earth and Life Studies; Board on Life Sciences; Health and Medicine Division; Board on Health Sciences Policy; Committee on Data Needs to Monitor Evolution of SARS-CoV-2. 2020. *Genomic Epidemiology Data Infrastructure Needs for SARS-CoV-2: Modernizing Pandemic Response Strategies*. Washington (DC): National Academies Press (US).
Nelson, Martha I., James O. Lloyd-Smith, Lone Simonsen, Andrew Rambaut, Edward C. Holmes, Gerardo Chowell, Mark A. Miller, David J. Spiro, Bryan Grenfell, and Cécile Viboud. 2019.

ACCEPTED MANUSCRIPT

"Fogarty International Center Collaborative Networks in Infectious Disease Modeling: Lessons Learnt in Research and Capacity Building." *Epidemics* 26 (March): 116–27.
Nicholls, Samuel M., Radoslaw Poplawski, Matthew J. Bull, Anthony Underwood, Michael Chapman, Khalil Abu-Dahab, Ben Taylor, et al. 2020. "MAJORA: Continuous Integration Supporting Decentralised Sequencing for SARS-CoV-2 Genomic Surveillance." *Cold Spring Harbor Laboratory*. https://doi.org/10.1101/2020.10.06.328328.
Oude Munnink, Bas B., David F. Nieuwenhuijse, Mart Stein, Áine O'Toole, Manon Haverkate, Madelief Mollers, Sandra K. Kamga, et al. 2020. "Rapid SARS-CoV-2 Whole-Genome Sequencing and Analysis for Informed Public Health Decision-Making in the Netherlands." *Nature Medicine* 26 (9): 1405–10.
Plessis, Louis du, John T. McCrone, Alexander E. Zarebski, Verity Hill, Christopher Ruis, Bernardo Gutierrez, Jayna Raghwani, et al. 2021. "Establishment and Lineage Dynamics of the SARS-CoV-2 Epidemic in the UK." *Science* 371 (6530): 708–12.
Rambaut, Andrew, Edward C. Holmes, Áine O'Toole, Verity Hill, John T. McCrone, Christopher Ruis, Louis du Plessis, and Oliver G. Pybus. 2020. "A Dynamic Nomenclature Proposal for SARS-CoV-2 Lineages to Assist Genomic Epidemiology." *Nature Microbiology* 5 (11): 1403–7.
Ranjan, Rajesh, Aryan Sharma, and Mahendra K. Verma. 2021. "Characterization of the Second Wave of COVID-19 in India." *bioRxiv*. medRxiv. https://doi.org/10.1101/2021.04.17.21255665.
Rockett, Rebecca J., Alicia Arnott, Connie Lam, Rosemarie Sadsad, Verlaine Timms, Karen-Ann Gray, John-Sebastian Eden, et al. 2020. "Revealing COVID-19 Transmission in Australia by SARS-CoV-2 Genome Sequencing and Agent-Based Modeling." *Nature Medicine* 26 (9): 1398–1404.
Seemann, Torsten, Courtney R. Lane, Norelle L. Sherry, Sebastian Duchene, Anders Gonçalves da Silva, Leon Caly, Michelle Sait, et al. 2020. "Tracking the COVID-19 Pandemic in Australia Using Genomics." *Nature Communications* 11 (1): 4376.
Singer, Joshua, Robert Gifford, Matthew Cotten, and David Robertson. 2020. "CoV-GLUE: A Web Application for Tracking SARS-CoV-2 Genomic Variation." *Preprints*. https://doi.org/10.20944/preprints202006.0225.v1.
Tegally, Houriiyah, Eduan Wilkinson, Marta Giovanetti, Arash Iranzadeh, Vagner Fonseca, Jennifer Giandhari, Deelan Doolabh, et al. 2021. "Detection of a SARS-CoV-2 Variant of Concern in South Africa." *Nature* 592 (7854): 438–43.
Thielen, Peter M., Shirlee Wohl, Thomas Mehoke, Srividya Ramakrishnan, Melanie Kirsche, Oluwaseun Falade-Nwulia, Nídia S. Trovão, et al. 2021. "Genomic Diversity of SARS-CoV-2 during Early Introduction into the Baltimore-Washington Metropolitan Area." *JCI Insight* 6 (6). https://doi.org/10.1172/jci.insight.144350.
United States. 1996. "Health Insurance Portability and Accountability Act of 1996. Public Law 104-191." *United States Statutes at Large* 110 (August): 1936–2103.
Van Noorden, Richard. 2021. "Scientists Call for Fully Open Sharing of Coronavirus Genome Data." *Nature* 590 (7845): 195–96.
Volz, Erik, Verity Hill, John T. McCrone, Anna Price, David Jorgensen, Áine O'Toole, Joel Southgate, et al. 2020. "Evaluating the Effects of SARS-CoV-2 Spike Mutation D614G on Transmissibility and Pathogenicity." *Cell*, November. https://doi.org/10.1016/j.cell.2020.11.020.
Volz, Erik, Swapnil Mishra, Meera Chand, Jeffrey C. Barrett, Robert Johnson, Lily Geidelberg, Wes R. Hinsley, et al. 2021. "Transmission of SARS-CoV-2 Lineage B.1.1.7 in England: Insights from Linking Epidemiological and Genetic Data." *bioRxiv*. medRxiv. https://doi.org/10.1101/2020.12.30.20249034.
Walensky, Rochelle P., Henry T. Walke, and Anthony S. Fauci. 2021. "SARS-CoV-2 Variants of Concern in the United States-Challenges and Opportunities." *JAMA: The Journal of the American Medical Association* 325 (11): 1037–38.
Washington, Nicole L., Karthik Gangavarapu, Mark Zeller, Alexandre Bolze, Elizabeth T. Cirulli, Kelly M. Schiabor Barrett, Brendan B. Larsen, et al. 2021. "Genomic Epidemiology Identifies

ACCEPTED MANUSCRIPT

Emergence and Rapid Transmission of SARS-CoV-2 B.1.1.7 in the United States." *medRxiv*, February. https://doi.org/10.1101/2021.02.06.21251159.

ACCEPTED MANUSCRIPT
